# Post-operative analgesic regime in a patient with congenital indifference to pain

**DOI:** 10.1093/jscr/rjaf275

**Published:** 2025-05-03

**Authors:** Thomas French, Sanjeet Singh Avtaar Singh, Emma Coley, Malcolm Will

**Affiliations:** Department of Cardiothoracic Surgery, Royal Infirmary of Edinburgh, Little France Crescent, EH16 4SA, Edinburgh, United Kingdom; Department of Cardiothoracic Surgery, Royal Infirmary of Edinburgh, Little France Crescent, EH16 4SA, Edinburgh, United Kingdom; School of Cardiovascular and Metabolic Health, University of Glasgow, 126 University Place, G12 8TA, United Kingdom; Department of Anaesthesia, Royal Infirmary of Edinburgh, Little France Crescent, EH16 4SA, Edinburgh, United Kingdom; Department of Cardiothoracic Surgery, Royal Infirmary of Edinburgh, Little France Crescent, EH16 4SA, Edinburgh, United Kingdom

**Keywords:** congenital indifference to pain, thoracic surgery, sarcoma, metastasectomy

## Abstract

A man in his thirties with congenital indifference to pain with anosmia (CIP) presented to a tertiary centre for an elective biopsy and video-assisted thoracoscopic surgery. Intra-operatively, he demonstrated no autonomic reflex in response to intubation or surgical stimulus, out of keeping with similar case reports in the medical literature. Post-operatively, he developed intermittent tachycardia, hypertension and pyrexia, which was controlled with opioid and non-opioid-based analgesics. Post-operative pyrexia and tachycardia alerted the patient to a developing chest infection, resulting in re-admission. Learning points include the importance of regular and accurate national early warning score documentation in patients with CIP, and the utility of conventional analgesia in tempering the autonomic response to stress in this patient group.

## Introduction

Congenital indifference to pain (CIP) or channelopathy-associated insensitivity to pain (OMIM #243000) is a vanishingly rare phenotype characterised by an absence of pain perception in affected individuals, often linked with olfactory disturbance. It is classified under subset 2D of the hereditary sensory and autonomic neuropathies (HSAN-IID).

Insensitivity to pain arises as a result of loss-of-function mutations in the gene encoding the voltage-gated sodium-channel unit Na_v_1.7 (*SCN9A*). Na_v_1.7 is expressed disproportionately in human nociceptive neurons and rat olfactory neurons [1], and selective deletion of *SCN9A* in rats results in anosmia and a lack of response to painful stimuli [2, 3]. Gain of function mutations of the gene result in ‘extreme pain’ syndromes such as primary erythermalgia and paroxysmal extreme pain disorder [4].

CIP is typically identified in early childhood, resulting in poorly healing wounds due to recurrent staphylococcal infection, accidental self-mutilation and recurrent bony fractures. Affected individuals might experience insensitivity to pain from birth, or it may develop later in life.

Estimating the prevalence of CIP and the larger subset of congenital analgesic phenotypes is difficult due to the scarcity and poorly classified nature of these conditions. Estimated prevalence of the broader HSAN-II subtype is only a few hundred patients worldwide [5]. Mentions of ‘congenital pure general analgesia’ first appear in the medical literature in 1932, when Dearborn described a 54-year-old man who had managed to shoot himself, stab himself and burn himself without eliciting any sort of pain response [6].

This case concerns a patient with CIP who presented for an elective operation. Due to the scarcity of true congenital analgesia, there exists no formal guidance for managing peri-operative analgesic pathways in this patient group. This case study discusses the approach taken to peri-operative analgesia in one such patient.

## Case presentation

A man in his thirties presented for an elective endoscopic ultrasound guided biopsy (EBUS) of a lesion at his right middle bronchus and video-assisted thoracoscopic surgery (VATS) lung resections for 2 nodules in his right lung.

**Figure 1:**
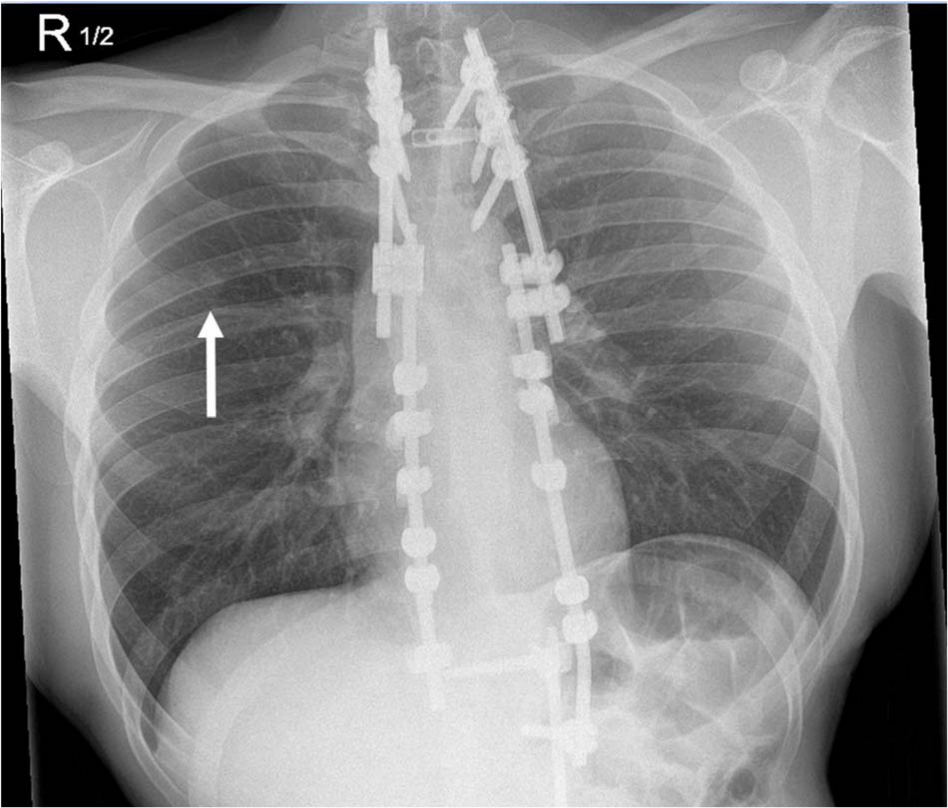
A monitoring radiograph 46 months after initial surgery noted a new nine-millimetre nodule in the right upper lung.

He had undergone excision and adjuvant radical radiotherapy of a pT3 N0 M0 sarcoma on the right-side of his neck 4 years earlier. Forty-six months after the original surgery, a routine chest radiograph identified a new nine-millimetre diameter right apical lung nodule (Fig. 1).

**Figure 2:**
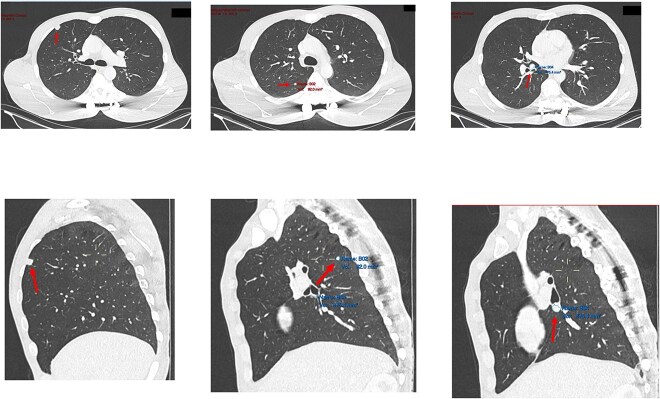
Transverse and saggital views of the three nodules identified on an investigative CT scan.

**Figure 3:**
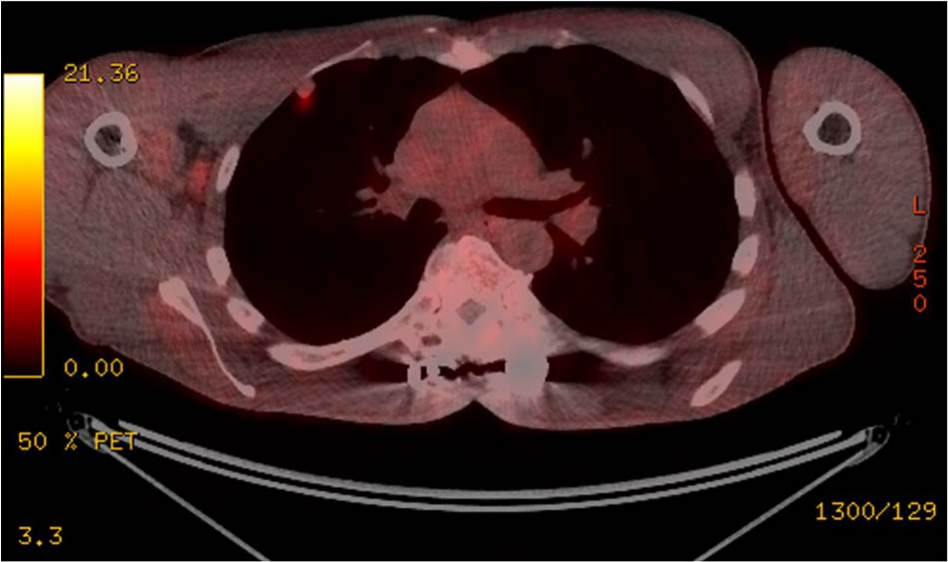
The pleural-based anterior right lobe lesion showed fluorodeoxyglucose uptake on a PET scan.

Subsequent computed tomography (CT) scan and positron emission tomography (PET) scan (Figs 2 and 3) confirmed the presence of three nodules in the right lung, suspicious for sarcoma metastases. An anterior, pleural-based upper lobe lesion, a posterior lower lobe lesion, and a lesion located at the bifurcation of his right middle and lower lobes.

His past medical history was notable for CIP with anosmia, and a host of related hospital presentations, including poorly healing ulcers, scoliosis, staphylococcus discitis, and infected soft tissue injuries. On one occasion, he had presented to the emergency department after feeling a painless ‘popping’ sensation in his left arm, and had been diagnosed with a biceps tendon rupture. Imaging showed widespread skeletal degeneration. His condition had first come to his attention after scalding himself whilst working as a plumber. He had not noticed the injury until later. Subsequent genetic sequencing revealed a diagnosis of CIP.

He had not required analgesia following his previous surgeries but had experienced autonomic responses to pain, characterised by tachycardia, hypertension, and pyrexia.

Clinical examination noted several small scald injuries affecting both the forearms and hands.

Following previous surgeries, he had not required treatment with a conventional analgesic regime and had suffered no consequent ill-effects. It was felt that to minimise the risk of an adverse autonomic response to pain, he should be treated using the department’s standard post-operative analgesic regime. This would involve patient-controlled boluses of an intravenous opioid immediately post-operatively, followed by paracetamol in conjunction with an ‘as required’ strong oral opioid, such as oxycodone or morphine, before step-down to a weaker oral opioid, such as codeine.

### Treatment

As the patient was not enthusiastic about the prospect of a lobectomy without a diagnosis, he underwent EBUS of the lesion located at the bifurcation of his right middle and right lower lobe and VATS wedge resections of the right upper lobe and right lower lobe.

Three samples were sent for histopathological analysis. The biopsied lesion, the upper lobe wedge, and the lower lobe wedge.

The patient displayed no autonomic response to the primary incision whilst under general anaesthetic: his blood pressure, pulse rate, and respiratory rate remained constant throughout the entire operation, from the induction of general anaesthetic.

Post-operatively, the patient was given an oxycodone patient-controlled analgesia (PCA) regime. He exhibited intermittent tachycardia, hypertension, and low-grade temperatures in the immediate post-operative period, which settled following administration of an opioid bolus. The PCA was stopped at 8 hours post-operatively as he was feeling nauseous after each bolus without any benefit.

As with the intravenous opioid, oral opioids and oral paracetamol in the immediate post-operative period settled the patient’s tachycardia, blood pressure, and pyrexia.

His chest drain was removed 1 day after the surgery, and he was discharged on post-operative Day 2.

### Outcome and follow-up

**Figure 4:**
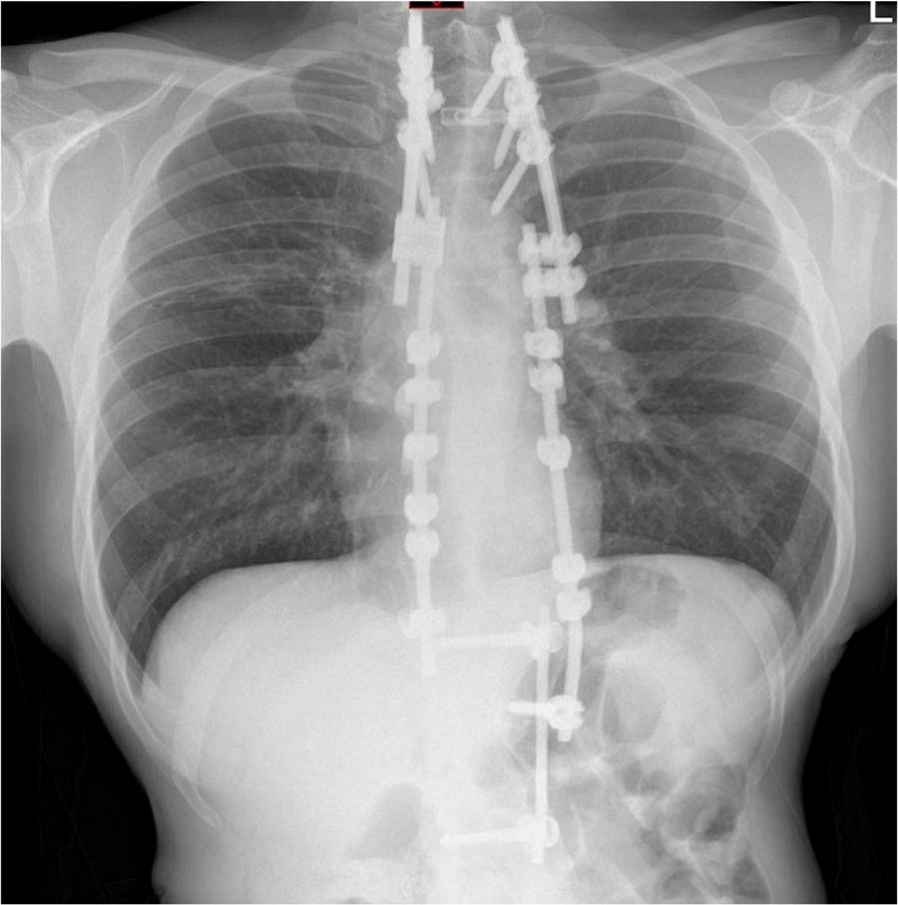
Repeat chest radiograph on re-admission to hospital with fevers and tachycardia.

Four days after the surgery, the patient was re-admitted due to fever, cough, and some small-volume purulent discharge from his drain site. A repeat chest radiograph (Fig. 4) was unremarkable, and a wound swab was negative. He was empirically treated with 24 hours of intravenous antibiotics and given a short 4-day course to complete at home. It was felt by the team that a lower threshold for treatment of infection would be appropriate, given his diagnosis of CIP. He was discharged 1 day after re-admission and made a good recovery.

Histopathology on the lesion biopsy at the bifurcation of the right middle and lower lobe bronchus was consistent with metastatic synovial carcinoma. The same was true for the nodule contained in the removed wedge of the right upper lobe. Histopathology for the lower lobe wedge was unremarkable.

He was referred back to the sarcoma multidisciplinary team and underwent radiation to the lower lobe nodule.

## Discussion

The authors identified sixteen reports concerning eighty-two patients in the literature that discussed the anaesthetic approach to patients with congenital analgesic syndromes. Of the reviewed reports, fourteen concerned patients with HSAN-IV (congenital insensitivity to pain with anhidrosis), and the remaining reports did not specify what subtype of congenital analgesia was under investigation.

Zlotnik *et al*. (35 cases) and Rozenstveig *et al*. (20 cases) noticed a high incidence of intra-operative bradycardia, hypotension and intra-operative cardiac arrest compared to the wider patient population [7, 8]. It should be noted, however, that HSAN-IV is associated with autonomic dysreflexia that has not consistently been observed in HSAN-II patients.

Majority of patients in the reviewed studies did not receive post-operative opiates, with tachycardia and pyrexia during airway manipulation and surgical stimuli noted in several cases [9, 10]. In only one other case was no cardiovascular response to surgical stimuli noted: a bilateral amputation of a 33-year-old man with an unspecified congenital analgesia [11].

Three of the patients identified were treated with no general anaesthetic or peripheral nerve block whatsoever, and went on to have an uncomplicated post-operative course [12, 13]. However, significant differences in pain response in the post-operative period were observed, and some of the patients observed actually developed pain in the post-operative or intra-operative period [14, 10].

This case study is notable for the fact that the patient did not display any autonomic reflex to pain throughout the operation, but did display an autonomic response to pain on several occasions in the post-operative period. This was well controlled with analgesics, which may have ramifications for patient comfort, although it is worth noting that the patient involved suffered side effects (nausea).

## Conclusions

CIP encompasses a wide range of overlapping and poorly understood phenotypes. Patient analgesic requirements are likely to vary significantly from patient to patient, but opioids can be useful in controlling the autonomic response to painful stimuli.
